# Transplacental Antibody Transfer: Mechanisms, Pregnancy-Related Disruptions, and Emerging Experimental Models

**DOI:** 10.3390/antib15010014

**Published:** 2026-02-06

**Authors:** Qiqi Li, Zhengyuan Huang, Zainab Saeed, Orene Greer, James A. Harker, Nishel M. Shah

**Affiliations:** 1 Department of Metabolism, Digestion and Reproduction, Imperial College London, 369 Fulham Road, London SW10 9NH, UK; 2 National Heart and Lung Institute, Imperial College London, Du Cane Road, London W12 0NN, UK

**Keywords:** transplacental IgG transfer, immunoglobulin G, neonatal Fc receptor, glycosylation, maternal immune cells, vaccination

## Abstract

The transplacental transfer of maternal immunoglobulin G from the mother to the foetus is central for providing immunity in early life, resulting in full-term newborns having IgG repertoires and levels similar to those of their mothers. The neonatal Fc receptor is recognised as the primary transporter of IgGs across the placental epithelium. Understanding the mechanisms of transplacental antibody transfer and factors that affect them is essential in optimising maternal vaccination strategies, ultimately protecting infants from various environmental pathogens. This review first outlines the biological mechanisms governing transplacental IgG transfer, followed by a discussion of how this process may be disrupted by physiological and pathological conditions during pregnancy, including preterm birth, hypergammaglobulinemia, maternal pathogenic IgG, maternal infections, hyperglycaemia, and exposure to biological therapies. We also summarise currently available models used to study transplacental IgG transfer, highlighting existing knowledge gaps and future directions for research in this field.

## 1. Introduction

**Figure 1:**
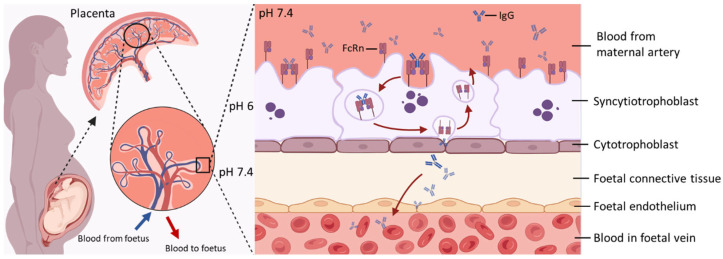
Key Features of Maternal–Foetal IgG Transfer via FcRn-Mediated Transcytosis. At the maternal–foetal interface, maternal IgG binds to FcRn expressed on the apical surface of SCT. The IgG–FcRn complex is internalised into endosomes, where acidic pH stabilises their interaction. This complex is then trafficked across the cell to the basal membrane. Upon exposure to physiological pH, IgG is released into the foetal stroma and subsequently enters the foetal circulation. After release, FcRn is recycled back to the apical membrane for reuse, enabling continuous transcytosis of maternal IgG during gestation. Created in https://www.BioRender.com.

At birth, newborns face exposure to numerous pathogens while possessing an immature immune system, which makes them especially susceptible to infections. Accordingly, maternal immunoglobulin G (IgG) begins to cross the placenta as early as 8–10 weeks of gestation, with the highest rate occurring in the third trimester (28–40 weeks of gestation), offering immune protection during the early stages of life [1,2,3]. Maternal plasma cells secrete antigen-specific antibodies essential for immune defence, among which IgA and IgG are predominantly responsible for protecting the newborn [4,5,6]. Only IgG however crosses the placenta to reach the foetal circulation, with different subclasses of IgG crossing the placenta with differential efficiency [4,7]. In the intervillous space, floating chorionic villi act as a barrier separating maternal and foetal circulations (Figure 1). They generally consist of a bi-layered epithelial structure with an outer multinuclear syncytiotrophoblast (SCT) layer and an inner core of mononuclear cytotrophoblasts, stroma containing fibroblasts and Hofbauer cells, and endothelial cells of the foetal capillaries [8,9]. There is general consensus that two receptors for the Fc region of IgGs, namely neonatal Fc receptor (FcRn) and Fcγ receptor III-A (FcγRIIIa), are co-localised on SCTs and considered to mediate the transplacental transfer of IgGs [10,11]. FcRn is considered as the primary transporter of IgGs across the placental barrier [12,13,14] while there is still considerable debate on the contribution of FcγRs in this process, with data from different laboratories showing evidence for [11,15] and against [12,16,17] direct FcγR involvement. Based on current knowledges, it is hypothesized that FcγRs fine-tune the kinetics of TPAT through several possible mechanisms: acting as a parallel transcytosis pathways independent from FcRn or co-receptors of FcRn [11,18,19], involving in multi-step mechanisms with FcRn [20], or immune complex clearance [21].

The transfer of IgGs through the SCT layer is achieved by fluid-phase pinocytosis, which comprises the pinocytic uptake of IgG by SCTs, fusion of vesicles containing IgGs with endosomes under a mildly acidic pH, and the release into stroma at a neutral pH [22]. Although the precise mechanisms that allow IgGs to cross both the villous stroma and foetal endothelium, and finally enter foetal circulation in vivo, are poorly elucidated, evidence suggests that alternative Fc receptors expressed on Hofbauer cells and the foetal endothelium might be involved in this process [18,23,24,25]. By the time the pregnancy reaches term, foetal IgG levels have surpassed maternal plasma IgG levels [3]. After birth, maternally derived IgG persists in the infant’s circulation and provides passive immune protection, typically lasting from approximately 6 weeks to 6 months, depending on factors such as the maternal immune status, antigen specificity, and IgG subclass or glycosylation profile [4,26,27,28].

Given the critical role of transplacental antibody transfer (TPAT) in providing immune protection to the foetus during pregnancy and to the newborn in the immediate period after birth, strategies to enhance immunity via the maternal–neonatal dyad have become a valuable public health practice [29]. The emergence of SARS-CoV-2 in early 2020 brought renewed attention to the safety and effectiveness of vaccination in pregnancy, reinvigorating clinical and scientific interest in maternal immunisation. Understanding the maternal factors that facilitate—or impair—optimal TPAT is therefore essential for maximising the success of this strategy [30,31]. A better understanding of the mechanisms of TPAT and the factors that affect them is crucial to optimize strategies of both maternal and neonatal vaccination. In this review, we first highlight the biological mechanism of TPAT and then discuss how this process may be altered in the context of various physiological and pathological challenges during pregnancy, including preterm birth, hypergammaglobulinemia, maternal pathogenic IgG, maternal infections, hyperglycaemia, and the use of immunotherapies. A comprehensive understanding of these factors can inform the development of maternal vaccination strategies, guide therapeutic interventions during pregnancy, and ultimately improve neonatal health outcomes. Currently available models of placental function to study the transplacental transfer of IgGs are also summarised in this review to highlight our knowledge gaps in this process.

## 2. Mechanisms of FcRn-Mediated Transport of IgG in the Placenta

Using FcγR/FcRn humanised mice to model maternal–foetal transfer, Borghi et al. found that FcRn, rather than FcγRIIIa, contributes to transplacental transfer of IgG [12]. Ex vivo human placental transfer models also highlighted the dominant role of FcRn in this process [14]. The engagement of IgG with FcγRs has been related to IgG-mediated inflammatory activities, while whether and how FcγRs expressed in placental cells contribute to the transplacental transfer of maternal IgG remain largely elusive [32,33]. In this review, we therefore focus on the latest research studying FcRn-mediated IgG transcytosis in physiological placentae.

The structural characteristics of IgG determine its interaction with FcRn in terms of binding manner, avidity, and distinct pH-dependant release. IgGs form a heterotetramer (comprised of two heavy and two light chain proteins covalently bonded by disulphide bridges), physiologically interacting with FcRn at a 1:2 ratio when imaged by electron microscopy [34]. The IgG population is further divided into four subclasses (IgG1–4) which exhibit different efficiencies in transplacental transfer (IgG1 > IgG3 > IgG2 ≈ IgG4) [4]. Although these four IgG subclasses are highly conserved, they differ in their constant region particularly in their hinges and upper CH2 domains [35]. Distinct structures among IgG subclasses might contribute to their different efficiencies in transplacental transfer because the FcRn engagement of IgG Fc region occurs at the CH3 and CH2 domain interface, which is involved with several residues (Ile253, Thr254, His310, His433, and His435) in the Fc region mediating various hydrogen-bonded and salt bridge interactions with FcRn [36,37]. This provides the structural basis for a pH-dependant switch as the FcRn-IgG interaction requires positively charged imidazole side chains on histidine, which occur at pH 5–6, while the binding is lost at pH 7.4 (Figure 1) as the side chains become neutrally charged [36,38,39]. Notably, FcRn plays a crucial role in prolonging the half-life of IgG by protecting it from lysosomal degradation, which accounts for the substantially longer serum persistence of IgG compared with other antibody isotypes [10,40]. Following pinocytosis by SCT, maternal IgG is internalised into endosomal compartments that subsequently acidify. The acidic environment promotes high-affinity binding of IgG to FcRn within these endosomes, thereby preventing its degradation and extending its half-life [10].

Through the development of therapeutic antibodies, the effects of post-translational modification on stability, half-life, and function of IgG have become increasingly clear [41]. Two commonly studied IgG modifications, glycosylation and methionine oxidation, have been shown to affect IgG activity [42,43]. IgG has a single N-linked glycan attached to Asn297 on each of the heavy chains of its Fc tail, largely occupying the cavity between two heavy chains [32,41]. An overall increase in both galactosylation and sialylation but decreased abundance of bisecting N-acetylglucosamine are observed in the Fc-glycan profile of maternal IgG with advancing gestation [16]. The N-linked glycans have been reported to alter both orientation and flexibility of CH2 domains, which eventually affects the Fc binding to the FcγRs backbone [44,45]. The removal of IgG glycans increases the flexibility of the FcRn binding site in the Fc region but leads to a small decrease in FcRn binding [46] while this effect is more pronounced in FcγRIIIa binding, where removal of the glycans almost completely disrupts receptor binding [47]. An analysis of ten pairs of foetal and maternal IgG samples concluded that the transplacental transfer of IgG is not Fc glycosylation-selective based on the fact that the profiles of Fc glycosylation of all the analysed IgG subclasses are largely comparable [16]. A more recent study by Borghi et al. also found no consistent difference in di-galactosylation of maternal and cord IgG1 (the most abundant IgG subclass) between two clinical cohorts from Uganda and Nicaragua [12]. Another recent study argued that IgG Fc glycosylation may contribute to the preference observed during TPAT, by fine-tuning receptor engagement within specific IgG subclasses. In particular, maternal IgGs with di-galactosylated Fc-glycans and ability to activate foetal NK cells are selectively transferred across the placenta to the neonate because of their enhanced bindings to both FcRn and FcγRIIIa [11], suggesting that antigen-specific Fc-glycan profiles ensure a preferential transfer. Further studies are required to reach a consensus on the role of IgG Fc region modification in modulating the transplacental IgG transfer. Additionally, about 10–20% of the Fab have N-glycosylation sites in the binding region [48,49]. IgG with Fab glycans has been called asymmetric antibody, and amounts increase during pregnancy [48,50]. A recent study in the context of the cell membrane has however suggested a suppressive role of IgG Fab glycans in FcRn-mediated transport [51]. The Fc fragment of IgG1 molecules also contains two conserved methionine residues at positions 252 and 428 that are subject to oxidation. These residues, which are located in the CH2 and CH3 domains, respectively, sit at the interface of the CH2/CH3 domains in the folded protein where FcRn binds [52]. Elevated levels of methionine oxidation on IgG1 were shown to increase its equilibrium dissociation constant for both FcRn and protein A [42], indicating reduced binding affinity. Using transgenic mice with human FcRn, Wang et al. further found that methionine oxidation of humanised IgG1 leads to a reduction in its half-life in the circulation, with the magnitude correlating well with the extent of methionine oxidation and changes in FcRn binding affinities [53]. Moreover, monoclonal antibodies possessing distinct Fab domains do exhibit different FcRn affinities even with an identical Fc region, which eventually affects their half-life [54,55]. These observations highlight that Fc modifications are not the sole determinants of IgG–FcRn interactions; rather, these interactions reflect whole-molecule effects that extend beyond the Fc region alone.

The expression of FcRn itself can also be modulated. Factors such as cytokines or infectious stimuli can either up- or down-regulate the expression of FcRn, and with binding sites for a wide range of transcription factors being found in the gene encoding for it, *FCGRT* [56,57,58,59,60]. There is some evidence that TPAT is limited by the potential saturation of placental Fc receptors at higher maternal IgG levels [29]. However, the association of placental FcRn expression and IgG transfer efficiency is still under debate because of controversial outcomes from different studies on both physiological and pathological placentae [4,15,61,62]; the controversy is potentially due to differential FcRn expression at different placental sites of collection and different manners of statistical analysis. Therefore, multicentre studies meeting both technical and interpretative integrity are required. More evidence is available indicating that the transmission efficiency of IgG subclasses varies due to differences in placental weight, birth weight, gestational age, and maternal antigen exposure [4,63].

## 3. Maternal Factors Affecting Antibody Transplacental Transfer

**Figure 2:**
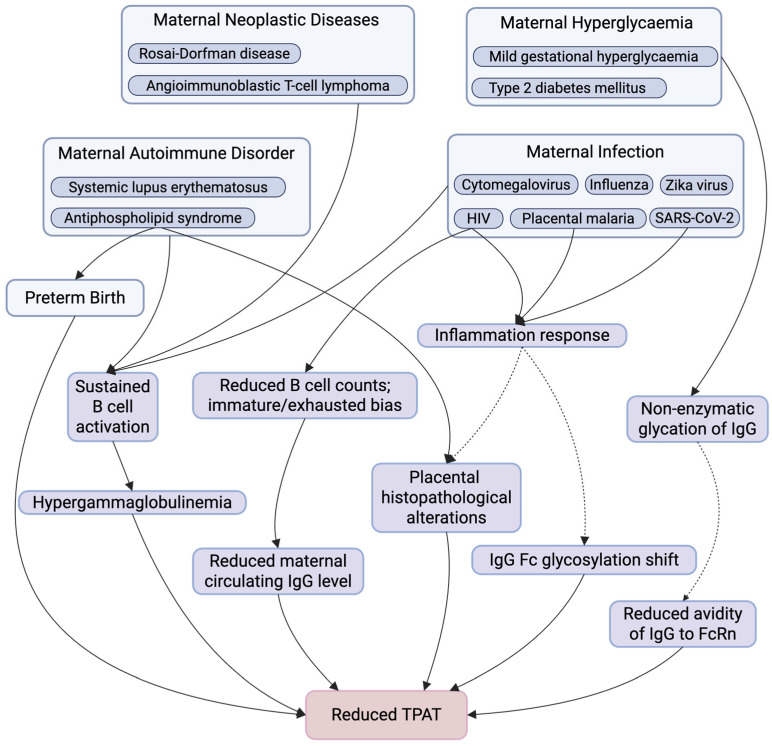
Summary of Maternal Factors Affecting TPAT. This figure summarises key maternal physiological and pathological conditions and downstream immunological and placental alterations that have been implicated in TPAT. Maternal conditions include autoimmune disorders, neoplastic diseases, infections, hyperglycaemia, and preterm birth, which may influence TPAT through multiple, potentially overlapping pathways. These include sustained B cell activation, altered B cell composition (with reduced total B cell counts and an immature or exhausted bias), hypergammaglobulinemia or reduced maternal circulating IgG levels, inflammatory responses, non-enzymatic IgG glycation, shifts in IgG Fc glycosylation, placental structure and function alterations, and reduce IgG–FcRn avidity, collectively contributing to impaired TPAT. Solid arrows indicate pathways with supportive evidence, whereas dashed arrows denote proposed or associative relationships that may not be direct, are context-dependent, or require further mechanistic investigation. Created in https://www.BioRender.com.

Given the critical role of TPAT in providing immune protection to both the foetus and the newborn, understanding the maternal factors that enhance or impair this process is equally essential [6]. Various maternal and placental conditions may be linked to influence the efficiency of TPAT, including hypertensive disorders of pregnancy (e.g., pre-eclampsia), placental insufficiency and intrauterine growth restriction, maternal obesity, smoking, inflammatory placental pathologies such as chorioamnionitis or villitis, and maternal malnutrition [64,65,66,67]. While these factors are clinically relevant, the available evidence remains limited and often indirect. Accordingly, this narrative review focuses on preterm birth, hypergammaglobulinemia, maternal pathogenic IgG, maternal infections, hyperglycaemia, as well as clinical interventions like immunotherapies (Figure 2) [68,69,70,71]. These factors were prioritised on the basis of more clinical evidence linking them to altered TPAT, most commonly assessed using cord-to-maternal antibody titre ratios (C/M ratios) [72,73,74]. A comprehensive understanding of these factors can inform the development of maternal vaccination strategies, guide therapeutic interventions during pregnancy, and ultimately improve neonatal health outcomes.

### 3.1. Kinetics of TPAT During Pregnancy

TPAT begins in the first trimester and increases exponentially as gestation progresses, a process that correlates positively with the placental FcRn expression throughout pregnancy. As early as 6 weeks’ gestation, small amounts of maternal IgG can reach the embryo and have been detected in the extraembryonic coelomic fluid and villous stroma [75]. Studies involving cordocentesis showed that by approximately 17 to 22 weeks of gestation, around 10% of maternal IgG concentrations are detectable in foetal circulation. The cord blood IgG concentration progressively increases from 17 weeks of gestation through late gestation and delivery, reaching around 50% of maternal levels by 28 to 32 weeks and eventually surpassing maternal levels between 37 and 40 weeks [3,64]. By full term, foetal IgG levels vary but are typically around 25% higher than those of the mother [76,77,78]. Since higher FcRn expression has been observed in placental tissue from 36 weeks of gestation onward, the transplacental transfer of IgG mostly occurs during the third trimester. Consequently, infants born prematurely, particularly those delivered before 32 weeks, have markedly reduced IgG concentrations relative to those born at term [61,76,79,80].

The kinetics of TPAT are therefore critical for optimising the timing of maternal vaccination. To ensure sufficient IgG transferred to the foetus, particularly in cases where preterm delivery is likely, vaccination during early pregnancy may be necessary. However, later administration may be preferable in some cases, as it aligns more closely with the peak period of IgG transferred across the placenta [81]. The optimal timing of vaccination during pregnancy varies depending on the specific vaccine. While current guidelines recommend maternal pertussis vaccination between 16 and 32 weeks of gestation [82], emerging evidence suggests that this window could be broadened, as optimal neonatal antibody levels and expected infant seropositivity can be achieved between weeks 13 and 33 [80]. Tetanus, diphtheria, and acellular pertussis vaccination between 30 and 33 weeks of gestation was associated with significantly higher geometric mean concentrations of anti-pertussis toxin IgG in infants at birth compared to vaccination at 20–24 weeks (27.3 IU/mL vs. 14.7 IU/mL), suggesting an improved TPAT with later gestational immunisation [4,83]. However, to achieve optimal cord blood antibody titres in both term and preterm infants requires scheduling pertussis vaccination at least 7.5 weeks prior to delivery. These findings support the administration of tetanus, diphtheria, and acellular pertussis vaccination during the second and third trimester, with earlier vaccination within this window recommended to provide comparable levels of protection for both term and preterm infants [84]. Similar principles apply to the respiratory syncytial virus (RSV) prefusion F protein maternal vaccine. Phase 3 data show that vaccination in the late second or third trimester reduces infant RSV-associated lower respiratory tract disease [85]. Accordingly, UK guidance recommends administration from 28 weeks’ gestation onwards (ideally at 28 weeks or soon after) to ensure adequate time for maternal antibody generation and efficient TPAT, including for infants born preterm [86,87]. Additionally, SARS-CoV-2 vaccination during the first trimester has been associated with higher C/M ratios compared to vaccination in later trimesters, suggesting that early immunisation can enhance passive immunity in neonates [88].

### 3.2. Maternal IgG Competition—Hypergammaglobulinemia

Normally, foetal IgG concentrations are positively correlated with maternal levels [7,64]. However, under pathological conditions where maternal IgG concentrations exceed 15 g/L, a condition defined as hypergammaglobulinemia, the limited capacity of FcRn becomes saturated. As a result, IgG molecules must compete for FcRn binding, and those that remain unbound are subject to cellular lysosomal degradation [64,89,90,91]. This saturation impairs the efficiency of transplacental IgG transfer and is associated with a negative correlation between maternal IgG levels and C/M ratios, observed for both total and antigen specific antibodies [4,92,93]. Hypergammaglobulinemia is characterised by the overproduction of multiple or a single immunoglobulin class, often driven by aberrant B cell activation, resulting in elevated levels of non-specific and/or functionally impaired antibodies, which can saturate the FcRn-mediated transport system, reducing the efficiency of transplacental transfer antigen-specific IgG [62,64,79,94,95,96]. Consequently, the quality of maternally derived IgG transferred to the foetus may be compromised, reducing the effectiveness of passive immune protection in the foetus and newborn.

Hypergammaglobulinemia is observed across a wide spectrum of pathological conditions. Transient hypergammaglobulinemia, often triggered by acute immune stimulation such as Epstein–Barr virus infection or *Plasmodium falciparum* in placental malaria (PM), can cause a temporary spike in maternal IgG levels [97,98,99]. While potentially detrimental to the shaping of the neonatal antibody repertoire, transient hypergammaglobulinemia may be avoidable or manageable through appropriate infection control and vaccination timing [100]. In contrast, chronic hypergammaglobulinemia is commonly associated with persistent immune dysregulation, including chronic infections such as cytomegalovirus and human immunodeficiency virus (HIV), as well as autoimmune conditions (e.g., systemic lupus erythematosus) and neoplastic diseases (e.g., Rosai–Dorfman disease, angioimmunoblastic T-cell lymphoma) [94,101,102,103,104]. These chronic states are characterised by sustained B cell activation and ongoing immunoglobulin overproduction, leading to long-term saturation of the FcRn pathway. Unlike transient forms, chronic hypergammaglobulinemia is difficult to modify during pregnancy, limiting the scope for intervention. Hypergammaglobulinemia may serve as a common underlying mechanism contributing to the impaired maternal–foetal IgG transfer observed in the context of various maternal infections or immune dysregulation.

### 3.3. Pathogenic Maternal IgG—Antiphospholipid Syndrome

Beyond antibody quantity, the qualitative properties of maternal IgG are equally critical in shaping placental function and pregnancy outcome, as illustrated by pathological settings in which IgG acts not as a passive mediator of neonatal protection but as an active driver of placental pathology. Antiphospholipid syndrome (APS) represents a paradigmatic IgG-mediated autoimmune disorder associated with obstetric morbidity and placental dysfunction, including recurrent miscarriage, late foetal loss, early-onset pre-eclampsia, foetal growth restriction, preterm birth, and placental insufficiency [105,106]. As such, APS may provide a clinically relevant model for examining how pathogenic maternal IgG may disrupt placental biology and TPAT. APS is characterised by circulating antiphospholipid antibodies, most notably IgG directed against β2-glycoprotein I (β2GPI), which exert direct effects on endothelial cells, trophoblasts and the coagulation system [107]. Beyond the classical laboratory criteria, the emerging concept of a “mosaic” of antiphospholipid antibodies, including non-criteria antibodies, has expanded the spectrum of pathogenic IgG species implicated in so-called seronegative APS, particularly in obstetric presentations [108]. Mechanistically, recent studies demonstrate that anti-β2GPI IgG can signal through LRP8/ApoER2 within lipid raft microdomains in endothelial cells, leading to impaired nitric oxide production and endothelial dysfunction, processes highly relevant to placental vascular homeostasis [109]. Although APS has not been directly linked to quantitative defects in TPAT, the profound alterations in placental inflammation, vascular integrity and Fc-dependent IgG signalling suggest that the placental microenvironment in APS may be suboptimal for efficient IgG transport.

### 3.4. Maternal Infection

With the increasing frequency of epidemics [110], pregnant individuals are at heightened risk of exposure to emerging infectious pathogens. Consequently, elucidating the impact of maternal infection on TPAT is essential for informing pandemic preparedness strategies and for optimising the protective effects of maternal vaccination and treatment on neonatal health outcomes. Different infections can act on distinct regulatory levels of the antibody transfer process, including maternal antibody quantity and quality, placental structure and functional integrity, and FcRn-mediated transport pathways [111,112,113,114,115,116,117]. Nevertheless, overlapping mechanistic themes can be identified across diverse infectious contexts, such as immune activation-driven inflammation, infection-associated placental pathology, and qualitative remodelling of the maternal IgG repertoire, including alterations in Fc glycosylation [112,113,114,115,116,117]. The specific combination of these factors, together with their timing during gestation, collectively determines the degree of impact on TPAT [118,119]. The following contents delineate how these mechanisms are differentially engaged in HIV infection, PM and SARS-CoV-2 infection during pregnancy. Table 1 summarises common infectious diseases that have been reported to affect maternal–foetal IgG transfer efficiency, along with their associated immunological alterations.

#### 3.4.1. HIV

With the widespread and effective use of antiretroviral therapy (ART), vertical transmission of HIV has significantly declined worldwide [125]. Nevertheless, in early infancy, neonates who are HIV-exposed but uninfected remain at a heightened risk for vaccine-preventable diseases relative to those unexposed to HIV [126,127]. This increased vulnerability is likely multifactorial, but one contributing factor is the impairment of TPAT [66,120,128]; at birth, HIV-exposed but uninfected neonates exhibit reduced C/M ratios for Haemophilus influenzae type b (23%), pneumococcus (15%), Bordetella pertussis (40%), and tetanus toxoid (27%) compared with HIV-unexposed counterparts [66].

Maternal HIV infection impairs transplacental IgG transfer by altering maternal B cell populations [120,121]. These immunological alterations are influenced by multiple factors and should be interpreted in relation to ART treatment status and the gestational timing of maternal infection. Patients with untreated HIV infection exhibit reduced total B cell counts in peripheral blood compared to uninfected individuals [111], which may impair antibody production. Additionally, chronic HIV infection is linked to a higher frequency of immature and exhausted B cells, which are less effective at generating antibodies [129]. As a result, HIV-infected pregnant women tend to have lower total antibody titre baseline, resulting in decreased levels of IgG specific to vaccines and pathogens being transferred via TPAT, thereby leaving infants with antibody levels below the protective threshold [121]. Fortunately, ART treatment has been shown to increase B cell counts and promote the normalisation of B cell subpopulations [111]. The extent to which these improvements can mitigate the HIV-induced reduction in maternal antibody production, however, remains dependent on the timing and efficacy of the intervention [102].

HIV infection is associated with persistent systemic inflammation, which drives antigen-specific remodelling of IgG Fc glycosylation. This glycosylation shift, characterised by reduced galactosylation, sialylation and fucosylation, is associated with enhanced FcγR binding and antiviral effector activity [122,130]. However, these modifications appear suboptimal for efficient TPAT. Consistent with this, Taylor et al. reported that HIV-infected pregnant women exhibited altered maternal IgG glycosylation profiles compared with uninfected counterparts, including higher proportions of agalactosylated and lower levels of digalactosylated and sialylated glycoforms. These glycosylation patterns were associated with increased binding to FcγRIIa and FcγRIIb but reduced transplacental transfer of vaccine-specific antibodies [112,113], indicating a trade-off between antiviral control and passive immunity conferred to the foetus.

#### 3.4.2. Malaria

PM, primarily caused by *Plasmodium falciparum* infection during pregnancy, is characterised by the sequestration of parasitised erythrocytes within the placental intervillous space [131]. These infected erythrocytes adhere to placental receptors, triggering inflammatory response. This immune activation is marked by the infiltration of maternal mononuclear cells and elevated levels of pro-inflammatory cytokines, which can lead to structural abnormalities in the placenta, including disruption of SCT integrity, thickening trophoblast basal lamina and increased syncytial knot formation [114,115,116,117]. This pathological environment has been linked to a significantly reduced efficiency of maternal IgG transfer. For instance, Okoko et al. reported a marked decrease in transplacental IgG transfer for herpes simplex virus 1 (69%), respiratory syncytial virus (58%), and varicella-zoster virus (55%), in malaria-infected placenta [62]. Similarly, multivariate analysis from western Kenya found that PM was significantly associated with reduced cord blood levels of Epstein–Barr virus antibodies, specifically anti-VCA-p18 and anti-EBNA1 [132].

#### 3.4.3. SARS-CoV-2

The angiotensin-converting enzyme 2 receptor, which facilitates SARS-CoV-2 cellular entry, has been identified in placental tissue, prompting initial concerns regarding potential viral replication within the placenta and consequent vertical transmission to the foetus [133,134,135]. Despite these concerns, current evidence indicates that transplacental transmission of SARS-CoV-2 is rare (0.63%) [119]. This limited vertical transmission is further supported by the absence of SARS-CoV-2-specific IgM antibodies in neonates, despite the presence of maternally transferred IgG [136]. In pregnancies affected by SARS-CoV-2 infection during the third trimester, placental pathological features such as maternal vascular malperfusion, including increased syncytial knots and focal perivillous fibrin deposition, are frequently observed [137]. These changes may reflect compromised placental function, potentially impairing the efficiency of transplacental IgG transfer [138].

Evaluation of TPAT following maternal SARS-CoV-2 infection has yielded mixed findings. Partey et al. demonstrated that levels of IgG specific to the SARS-CoV-2 nucleoprotein (N-protein) and spike receptor-binding domain in cord blood strongly correlate with maternal antibody concentrations (N-protein: R_s_ = 0.7155, *p* < 0.001; receptor-binding domain: R_s_ = 0.8693, *p* < 0.001), indicating effective TPAT that confer passive immunity to the neonate [139]. Furthermore, a reported mean C/M ratio of 1.27 (95% CI: 0.69–2.89) suggested no significant difference in TPAT efficiency based on maternal disease severity [119]. However, a comparative analysis suggests that hospitalised patients, who typically exhibit more severe symptoms, develop higher antibody titres than mildly symptomatic or asymptomatic individuals [140], which may potentially imply an enhanced efficiency of TPAT in severe maternal cases [119]. This hypothesis is supported by findings demonstrating significantly higher C/M ratios in mothers who experienced severe to critical illness compared to those with mild or no symptoms [141].

Furthermore, a positive correlation has been established between TPAT efficiency and the time interval between maternal infection and delivery. Maternal SARS-CoV-2 infection in late pregnancy (third trimester) is associated with measurably reduced placental transfer of SARS-CoV-2-specific antibodies compared with other vaccine- or pathogen-specific antibodies, potentially linked to acute inflammation-induced alterations in Fc glycosylation [118]. Maternal infections occurring during the early trimesters (first and second) were associated with more effective antibody being transferred to the foetus [119]. Importantly, the efficiency of TPAT observed in vaccinated pregnant individuals is comparable to that seen in those with natural infection [142]. These findings collectively support the administration of SARS-CoV-2 vaccines during early pregnancy as a strategy to enhance neonatal protection via passive immunity. This recommendation is further reinforced by evidence from Atyeo et al., in which median C/M ratios for SARS-CoV-2-specific IgG were approximately 1.5, 1.3, and 1.0 after first-, second-, and third-trimester vaccination, respectively, consistent with greater TPAT efficiency with earlier immunisation [88]. The insights gained from maternal SARS-CoV-2 immunisation also offer valuable guidance for optimising vaccination strategies against other emerging infectious diseases during pregnancy.

### 3.5. Maternal Hyperglycaemia

Hyperglycaemia during pregnancy, including conditions such as gestational diabetes mellitus and type 2 diabetes mellitus (DM-2), is increasingly prevalent worldwide and may significantly affect the efficiency of TPAT [143]. Maternal hyperglycaemia alters both maternal systemic immune regulation and placental histology, thereby influencing IgG being transferred to the foetus through various mechanisms.

Maternal and cord blood IgG levels vary depending on the type and severity of glycaemic disturbance. In women with mild gestational hyperglycaemia (MGH), maternal IgG levels are comparable to those in normoglycaemic pregnancies, while levels are significantly reduced in individuals with DM-2. Despite this, cord blood IgG concentrations remain similar across all groups [144]. This discrepancy suggests that compensatory or alternative mechanisms may preserve foetal IgG acquisition even in more severe diabetic conditions, though the underlying mechanisms remain to be fully elucidated. Structural changes in the placenta may contribute to altered transplacental IgG transfer in hyperglycaemic pregnancies. In cases of MGH, increased placental villous capillarisation and a higher number of SCT have been reported, which may facilitate TPAT and the movement of other macromolecules across the placenta [145,146]. While total IgG C/M ratios do not significantly differ between normoglycaemic and hyperglycaemic pregnancies, subclass-specific analyses reveal more nuanced differences. In the DM-2 group, transfer ratios of IgG1, IgG3, and IgG4 are reduced, suggesting impaired subclass-specific transfer. In contrast, in the MGH group, the ratio of IgG3 was increased compared to normoglycaemic controls, indicating that mild hyperglycaemia may differentially modulate IgG subclass trafficking [144].

Moreover, in diabetes mellitus, elevated circulating glucose levels can lead to non-enzymatic glycation of IgG molecules, potentially altering their structure and function [147,148]. These glycation-induced modifications have been hypothesised to reduce the binding avidity of IgG to FcRn, thereby impairing its transplacental transfer [65]. However, evidence remains inconclusive. For instance, Goetze et al. experimentally generated highly glycated IgG1 and IgG2 and assessed their binding to various Fc receptors, including FcγRIIIa and FcRn. Their findings indicated that glycation does not significantly affect receptor-binding affinity [149], challenging the assumption that glycation disrupts FcRn-mediated transport. Additionally, a study reported significant associations between maternal glucose homeostasis markers (e.g., HOMA2-IR, HOMA2-%B) and specific IgG glycosylation traits. Yet, no significant differences in overall IgG N-glycosylation patterns are detected between women with and without gestational diabetes mellitus [150]. This discrepancy may reflect the complex physiological adaptations during pregnancy that obscure subtle glycosylation-related effects, underscoring the need for more refined longitudinal analyses.

### 3.6. Transplacental Transfer of Bioengineering IgG—Pregnant Women Require Immunotherapies

The number of effective monoclonal antibody (mAb) biologics used to treat immune-mediated inflammatory diseases and cancer is increasing [151,152]. Like the endogenous IgG, those mAb biologics applied in immunotherapies that are necessary to manage maternal health can interact with FcRn, facilitating their transplacental transfer to foetal circulation during pregnancy [153]. In most cases, mAb biologics with modifications of their binding site with FcRs have a prolonged half-life and higher avidity with FcRn [154,155,156], but the impact on transplacental transfer of conventional antigen-specific antibodies has not been well elucidated, and their safety in relation to foetal development has become a significant concern. A study evaluating the amount of transplacental transfer observed between IgG1 mAb products treating inflammatory bowel disease on the day of birth confirmed the TPAT of IgG1 subclass mAbs (e.g., infliximab, adalimumab, and ustekinumab) targeting tumour necrosis factor alpha (TNFα), IL-12 and IL-23 [157]. Infants exposed to rituximab (an anti-CD20 mAb) in utero have been reported to exhibit the down-regulation of both B cell numbers and circulating IgG levels, as well as potential infection complications [158,159,160]. Accordingly, modifications of some mAb biologics have been used to avoid TPAT. For instance, Certolizumab, an anti-TNFα mAb, lacks an Fc portion, making it unable to bind to FcRn, and is considered safe to use throughout pregnancy; another anti-TNFα mAb, Etanercept, which possesses a modified Fc portion, also exhibits a low capacity to cross the placenta, and is not associated with an increased risk of foetal malformations, preterm delivery or pregnancy [161,162,163]. There has also been an enormous expansion in the development of engineering strategies involving FcRn to modulate the dynamic behaviour of antibodies, antigens and albumin, showing considerable potential for use in the clinic [164]. Novel strategies have potential in striking the balance between maternal health management and foetal harm minimalisation.

Furthermore, concerns about if early exposure to mAb biologics in utero affects the timing of neonatal vaccination and the effectiveness and safety of vaccination administered to neonates have also been raised [165]. Neonatal B cell depletion has been reported in pregnancies exposed to rituximab; however, none of these neonates experienced infectious complications or adverse reactions to vaccination, and B cell counts normalized in all cases within six months [166]. In contrast, early exposure to belimumab, a BAFF neutralising mAb, in utero is reported to be significantly associated with neonatal infections [167]. The safety of vaccinations during the first year of life in infants exposed to biologics in utero has also been evaluated in a systematic study [168], highlighting that the incidences of adverse event are different among vaccination (e.g., Bacillus Calmette–Guérin vaccine, rotavirus vaccine, and measles–mumps–rubella vaccine). Therefore, therapeutic strategies should be individualised accordingly to ensure the safety of both mother and infant. Although TNF inhibitors (TNFis) are commonly used biologics for immune-mediated inflammatory diseases and considered to be essentially safe for the foetus during pregnancy [169], the continued therapy of TNFis during late pregnancy is reserved only for patients with active disease, considering their possible effects on neonatal infection risk and vaccination schedules [170]. A recent study suggested that treatment with TNFis in pregnant patients with rheumatic disease induces lower maternal anti-pertussis IgG antibody levels in newborns in spite of no major differences in IgG antibody levels upon maternal tetanus-diphtheria-and-acellular-pertussis vaccination in pregnant women with or without immune-modulating treatment. As for neonates born to mothers who were treated with rituximab or belimumab during pregnancy, close monitoring of B cell counts and serum biologic levels after birth are recommended to facilitate early detection and management of potential infections [170]. The European Alliance of Associations for Rheumatology recommends that infants exposed to biologic agents before 22 weeks of gestation can follow the standard vaccination schedule, including live vaccine, while those exposed to biologics during the second and third trimester are advised to avoid the administration of live vaccines within the first six months of life [171].

## 4. Future Perspectives on Models of Placental Function Available to Study IgG Transplacental Transfer

### 4.1. Murine Model

Despite that marked differences between human and mouse placentae [172,173], in terms of unveiling the mechanism of FcRn-mediated transport of IgG, the direct masking or mutating of human FcRn in vivo is challenging, making murine models a valuable and appealing alternative. Transgenic murine models such as mice lacking endogenous FcRn but expressing a human FcRn transgene (Table 2) are currently proving to be a productive model for the assessment of FcRn-mediated transport of IgG [12], safety of vaccination for pregnant women [174], or potential therapeutics using bioengineering IgG molecules [175] because mouse FcRn can be bound to by IgGs from many different species with high affinity [176].

### 4.2. Placenta-Originated Model

In the meantime, diverse in vitro and ex vivo models of placental function relevant to humans or derived from human placental tissue (Table 2), such as primary cells or cell lines of placental origin, organoid, placental explant, or placental perfusion, have also been intensively developed to advance the research on transplacental transfer of IgG and other substances over the years, of which the critical evaluation has been previously reviewed [185,186]. Although human placental perfusion models more closely simulate the functional units of term placental tissue than cell monolayers or placental explants, it is also necessary to consider how morphological changes and increasing FcRn expression during placental development [3,61] influence the transplacental transfer of IgG.

### 4.3. Computational Modelling

The development of novel techniques, for example, a quantitative mechanistic model to unveil the determinants of transplacental transfer of IgG [187] or using the ‘placenta-on-a-chip’ model combining microfluidics and diverse human placental cells in 3D structures to study placental translocation and inflammatory responses [188,189], is providing more insights into understanding the transplacental transfer of IgG. Wessel and Dolatshahi employed a quantitative mechanistic model to reveal several key factors that contribute to IgG inter-subclass competition and potentially inter- and intra-patient IgG transfer heterogeneity [187]. Filippo et al. developed a low-dimensional machine learning model compiling a database of 248 compounds with experimental information about their placental transfer, which is considered to be useful in predicting placental drug transfer during pregnancy [190].

### 4.4. Strengths and Limitations

Compared to the murine model and in vitro culture of cell monolayer culture, the primary advantage of the perfusion model is its preservation of tissue functional integrity and cellular barrier function. Notably, significant differences in IgG processing have been observed between in vitro cell culture systems and placental perfusion models, and gestation-dependent transplacental IgG transfer in humans has been demonstrated beginning in the first trimester [17,191]. A recent review has offered important insights into the role of dual ex vivo perfusion models in investigating IgG transfer, while critically evaluating the various placental perfusion methodologies [192]. Nevertheless, despite their advantages, these models are limited by their inability to recapitulate the longitudinal placental development and the progressive accumulation of maternal antibodies throughout gestation. Mathematical modelling, as an emerging methodological approach, is therefore leveraged to elucidate nonlinear dynamics across diverse physiological systems and to inform the rational design of therapies and vaccines. Such models allow the concurrent analysis of multiple interdependent components, highlight the critical role of temporal dynamics, and integrate regulatory structures such as feedback loops [187]. Furthermore, mathematical modelling can better identify kinetic constraints and provide a quantitative framework in the context of TPAT for evaluating those relative factors governing this special transplacental transfer.

## 5. Conclusions

Pregnancy and early life represent periods of pronounced immunological complexity. Maternal adaptations maintain foetal tolerance while preserving host defence, and newborns’ immunity depends largely on the acquisition of maternal IgG [4,7,193]. TPAT therefore constitutes a critical conduit of immunity across the maternal–neonatal dyad, offering protection both in utero and in the immediate postnatal period, and serves as the biological basis for maternal vaccination strategies [1,2,3]. Consequently, optimising maternal immunisation to enhance TPAT has become an increasingly important public health priority [29]. The efficiency of TPAT varies and can be affected by multiple clinical and physiological factors, including preterm birth, hypergammaglobulinemia, maternal pathogenic IgG, maternal infection, hyperglycaemia, and immunomodulatory therapies [68,69,70,71]. These observations indicate that key determinants of IgG transfer remain insufficiently defined, particularly with respect to how maternal immune status, placental transport pathways, and antibody structural features collectively influence transfer across the SCT. Recent technological advances offer potential avenues to address these gaps. Fc-engineered IgG molecules generated through rational design exhibit extended half-life and enhanced therapeutic efficacy [194]. Such bioengineering approaches may be leveraged to improve passive immunity in newborns, especially as understanding increases regarding how molecular and cellular regulators govern the selective transfer of antigen-specific antibodies. Current understanding of FcRn–IgG immunobiology indicates that blocking FcRn represents a promising and effective approach to lowering circulating pathogenic IgG autoantibodies and mitigating IgG-driven diseases. Multiple FcRn inhibitors that specifically disrupt IgG recycling are now advancing swiftly toward clinical application in both neurology and haematology [195], exhibiting potential for future use in a much wider variety of antibody-mediated autoimmune diseases rather than optimising maternal and neonatal vaccination only. In parallel, although the inadequacy of existing experimental models was once considered to hinder the investigation in dynamics of IgG transfer in pregnant women and the rational design of maternal vaccines, computational modelling or in silico mechanistic models in combination with experimental data has now emerged as a powerful strategy to dissect the process regulating TPAT [196]. Continued progress in these areas is likely to support the development of maternal vaccines and antibody-based interventions that more consistently achieve protective immunity in both pregnant women and their infants.

## Figures and Tables

**Table 1:** Impact of maternal infections on TPAT efficiency.

Infection	Maternal Infection-Associated Alterations	References
HIV	Alters maternal B cell subsets, reduces maternal IgG levels and induces IgG glycosylation shift compromising IgG binding to FcRn	[112,120,121,122]
Malaria	Induces local placental inflammation and leads to histopathological alterations in placental structure	[114,115,116,117]
SARS-CoV-2	TPAT efficiency is positively correlated with the interval between maternal infection and delivery	[119]
Influenza	Impairs maternal antibody quality with potential downstream effects on TPAT efficiency	[123]
Zika virus	Causes placental infection and barrier disruption based on mechanistic studies in mice	[124]

**Table 2:** Murine and human models used for the investigation of transplacental transfer of IgG.

Name	Species	Model Type	Target of Interest	Properties	References
Transgenic mice deficient in FcRn	Mice	In vivo	FcRn function	FcRn^−/−^ foetuses showed negligible IgG concentrations.	[177]
Fc receptor humanized mice	Mice	In vivo	FcγR or FcRn function	Deficient in mouse Fc receptors and expressing human Fc receptors as a transgene.	[12,53,176]
Choriocarcinoma- derived cell line BeWo	Human	In vitro	SCT layer	Cells that can be cultured to form polarized, confluent monolayers with tight junctions.	[178,179,180]
Trophoblast organoids	Human	In vitro	SCT layer	Human trophoblast stem cells form spherical organoids with a single outer layer of SCT cells that display a barrier function.	[181]
Placental transfer model	Human	Ex vivo	The binding affinity of antitumour monoclonal antibodies to FcRn/FcγR	Cannulated and re-perfused term placental cotyledon.	[182]
First-trimester placental villous explants	Human	In vitro	The internalization of antiphospholipid antibodies by SCT cells	Placental explants of 10–30 mg wet weight were cultured in culture media supplemented with different antibodies.	[183]
Recombinant IgG antibodies lacking effector functions	Human	In vitro	The strategy to control the transplacental transfer of pathogenic maternal IgG	Human IgG3 antibodies are devoid of FcgR and C1q binding, cellular cytotoxicity and complement activation but retain FcRn binding.	[17]
Engineered IgG1 antibody	Human	Ex vivo	The activity of the engineered human IgG1 in both human and murine systems.	IgG1 mutants (H433K and N434F) transferred across human placenta more efficiently than wild-type IgG1.	[13]
M281, a fully human, aglycosylated monoclonal IgG1 anti-FcRn antibody	Human	Ex vivo	Blockage of FcRn for inhibition of transfer of pathogenic IgG antibodies	M281 significantly decreases the transfer rate of adalimumab across dually perfused human placental lobule.	[184]

## Data Availability

No new data were created or analysed in this study.

## Abbreviations

IgG	Immunoglobulin G
FcRn	Neonatal Fc receptor
TPAT	Transplacental antibody transfer
SCT	Syncytiotrophoblast
FcγRIIIa	Fcγ receptor III-A
C/M ratios	Cord-to-maternal antibody titre ratios
RSV	Respiratory syncytial virus
PM	Placental malaria
APS	Antiphospholipid syndrome
β2GPI	β2-glycoprotein I
HIV	Human immunodeficiency virus
ART	Antiretroviral therapy
N-protein	Nucleoprotein
DM-2	Type 2 diabetes mellitus
MGH	Mild gestational hyperglycaemia
mAb	Monoclonal antibody
TNFα	Tumour necrosis factor alpha
TNFi	Tumour necrosis factor alpha inhibitor
